# *Trichinella* spp. in Slaughtered Pigs of India: From Neglected Disease to an Emerging Food Safety Threat for Public Health

**DOI:** 10.1155/2024/7550006

**Published:** 2024-01-23

**Authors:** Deepali Gopal Kalambhe, Harpreet Kaur, Jatinder Paul Singh Gill

**Affiliations:** ^1^Centre for One Health, Guru Angad Dev Veterinary and Animal Sciences University, Ludhiana, Punjab, India; ^2^Directorate of Research, Guru Angad Dev Veterinary and Animal Sciences University, Ludhiana, Punjab, India

## Abstract

**Background:**

In India, *Trichinella* nematode worm remains highly neglected due to the paucity of research. Recent trichinellosis outbreaks in humans associated with pork meal in north India have highlighted an emerging food safety concern due to this neglected parasite.

**Aim:**

This study aimed to ascertain the existence of *Trichinella* species and to identify them in slaughtered pigs intended for human consumption in the Punjab and Uttaranchal regions of North India.

**Materials and Methods:**

A total of 1,194 slaughter pig tongue samples were screened in 239 pools using the double separatory funnel method for recovery of *Trichinella* larvae. The species of recovered larvae were confirmed by multiplex PCR assay and sequencing. A phylogenetic tree was constructed to ascertain the closest lineage.

**Results:**

This study reported 3.77% and 1.26% *Trichinella* positivity in pooled and individual pig samples, respectively. Pigs slaughtered in Punjab reported higher positivity (1.73%) than pigs in Uttaranchal (0.81%). Among all places, Jalandhar recorded highest positivity of 5.66%, followed by 2.22% in Nainital and 0.8% in Patiala, whereas none of the pig samples from Chandigarh, Ludhiana, Bathinda, Tehri Garhwal, Pauri Garhwal, Kashipur, Haridwar was positive. Molecular confirmation by PCR and sequencing confirmed the recovered *Trichinella* larvae as *T. britovi* and *T. nelsoni. Conclusion. Trichinella* parasite remained highly neglected in India; however, the detection of sylvatic *Trichinella* species in pigs intended for human consumption indicated the emergence of zoonotic foodborne risk at wild animal-domestic pig and human interfaces. To the best of our knowledge, this is the first study reporting *T. britovi* and *T*. *nelsoni* in pigs of North India. *Trichinella* positivity in slaughtered pigs is an early alarm for the emergence of foodborne risk from this neglected parasitic worm. Considering the importance of pigs as a source of meat and their role as a reservoir for *Trichinella* raised the food safety concern that warrants strict meat inspection and extensive studies on neglected parasites in India.

## 1. Introduction

Pork is implicated to cause trichinellosis in man due to the presence of microscopic nematode worms of *Trichinella* species. In the true sense, the term “trichinellosis” is an exclusively meat-borne disease in humans, acquired by consumption of raw or inadequately cooked infected meat, especially pork. Pork from pigs infected with muscle larvae is the commonest source of human trichinellosis; however, the persistence of the parasite in wild animals via sylvatic cycles and the traditional practice of consuming inadequately cooked meat is imperative in the epidemiology of trichinellosis worldwide.

Genetically, *Trichinella* has been classified into 10 species, including a recently identified *T. chanchalensis* (T13) and three genotypes within two clads, capsulated and noncapsulated. Globally, *T. spiralis* is a classical agent of human trichinellosis, whereas *T. britovi* is seldom associated with mild asymptomatic infections [1]. Documented facts have highlighted the mortality with *T. spiralis*, *T. nelsoni*, *T. nativa*, *T. pseudospiralis*, and *T. murrelli* in infected individuals [2, 3]. It is believed that the larvae of *Trichinella* nematodes, especially *T. britovi*, can survive in frozen pork for up to 3 weeks and in the muscles of carnivores for up to 11–12 months [3]. Further, studies have reported higher resistance of *T. nativa* to freezing and survival in the muscles of carnivores for up to 5 years [4]. However, cooking the meat core at 65°C for 1 min kills the *Trichinella* larvae.

For preventing the incidence of trichinellosis, especially from pork, a compulsory veterinary meat inspection was introduced during the 19th century following an outbreak of trichinellosis in Europe [3]. Hitherto, in India, the risk of trichinellosis to pork consumers was generally overlooked and poorly investigated. Trichinellosis has rarely been documented in India. This could be due to the hypothesized lower prevalence (0.4%–0.6%) of *Trichinella* species in pigs of India as reported in previous surveys [5, 6]. Some older reports have documented the presence of encapsulated *Trichinella* larvae in domestic cats, wild toddy cats, wild civets, rodents, and domestic pigs [7–12] and nonencapsulated species in Indian mole rats [13]. Some latest studies from Maharashtra and Punjab have also reported less prevalence (0.69%–0%) in slaughtered pig samples collected from Goa, Gujarat, Assam, Chandigarh, Punjab, and Uttarakhand [14–16]. The incidence of human trichinellosis is underestimated in India due to nonspecific symptoms, lack of clinical suspicion, and nonavailability of diagnostic laboratory facilities. Until an outbreak of human trichinellosis in the year 2011, there was only one documented case of trichinellosis from India in women with a history of pork consumption [17]. Only a few sporadic outbreaks from a hilly state of Uttaranchal between 2009 and 2011 [18], a rare case of human trichinellosis with pyomyositis and secondary osteomyelitis from PGIMER, Chandigarh [19], a case of trichinellosis associated miscarriage [20] and *T. spiralis* associated squamous cell carcinoma from Dehradun have been reported recently [21], which all were linked to the history of consumption of pork of wild boar or pork products.

Nevertheless, with the growing pig farming in India, it is a need of time to explore the epidemiological distribution, ecology, and prevalence of *Trichinella* species in pigs of India to ensure food safety. Trichinellosis is an emerging, still neglected zoonosis in India. Limited studies from India, however, confirmed the cases of human trichinellosis [22, 23]. The latest outbreak in Uttaranchal claimed the lives of people who relished semi-cooked pork feasts [24]. Trichinellosis is a 100% food-borne disease that can be easily prevented by strict compliance with meat inspection and food safety measures.

Considering the role of pork in frequent sporadic outbreaks in humans in Uttaranchal, the paucity of documented evidences of *Trichinella* in the pig population, and the looking into the growing demand for pig farming in Punjab, it is imperative to study this parasite in pigs (domestic and wild) slaughtered for human consumption in these regions. The important risk for human trichinellosis is unhygienic and clandestine slaughtering without any meat inspection. Even at government-approved slaughterhouses, meat samples are never screened for the presence of *Trichinella* larvae unlike it is mandatory in other developed countries.

Acknowledging the public health importance of trichinellosis associated with pork consumption and the absence of studies identifying the prevalent *Trichinella* species, the present investigation was envisaged to ascertain the existence of *Trichinella* species in slaughter pigs from selected districts of Punjab and Uttaranchal states in north India.

## 2. Materials and Methods

### 2.1. Study Population

The present study targeted the pigs slaughtered and intended for human consumption in the states of Punjab and Uttaranchal and the union territory, Chandigarh. Samples were collected from the local slaughtershops (unorganized) and from the organized slaughterhouses located in the selected districts of these regions. Pigs slaughtered in Uttaranchal were mostly backyard pigs, and occasionally, wild pigs were also slaughtered by local butchers, whereas organized slaughterhouses in Punjab and Chandigarh slaughtered both intensively reared pigs and backyard pigs. Unorganized local butcher shops slaughtered mostly free-ranged pigs.

### 2.2. Collection, Transportation, and Storage of Samples

This research was conducted between 2017 and 2020. A total of 1,194 pig tongue samples were collected from selected places in Punjab and Uttaranchal states in North India (Table 1). The samples were collected from various slaughtered houses/shops in Ludhiana, Jalandhar, Bathinda districts of Punjab state and Tehri Garhwal, Pauri, Garhwal, and Haridwar districts in Uttaranchal state of India. The samples were transported to the laboratory by maintaining a proper cold chain on ice packs and were processed fresh or stored at −20°C until processed.

### 2.3. Tissue Digestion Assay for the Recovery of *Trichinella* Larvae

The pooled tongue muscles were subjected to artificial digestion using the double separatory funnel method for the recovery of *Trichinella* larvae as per the methods recommended by the OIE, European Union Reference Laboratory for Parasites [25]. Briefly, 2,000 ml of potable water was pre-warm to 46°C in a 3-l capacity glass beaker kept inside an incubator. The 100 g of pooled tongue sample (20 g each from five individual tongues) was cleaned by removing the fascia, fat, and papillae. The cleaned tongue was cut into small pieces, finely minced, and grinded with 20 g pepsin (1 : 10,000 National Standard Formulary strength, HiMedia). Ground sample paste was added to 2,000 ml of prewarmed 1% acidified water (calibrated with conc. HCl) at 46°C in a 3-l capacity glass beaker. The mixture was incubated in a shaker incubator preset at 46°C at 100 rpm for 1 hr or until the samples were completely digested. After digestion, the mixture was sieved (mesh size approximately 180–200 *µ*m) into a separatory funnel of 3-l capacity and was allowed to sediment for 30 min without disturbing. After 30 min of primary sedimentation, 50 ml of sediment was released into the second separatory funnel (500 ml capacity) connected below the opening of the first separator funnel. This 50 ml of sediment was diluted by adding 450 ml of prewarmed (46°C) potable water and allowed to sediment undisturbed for 15 min. After 15 min, approximately 20 ml of sediment was collected in a gridded transparent petri dish and kept undisturbed for 5 min to let the larvae settle down. The sediment in the petri dish was observed under 10x magnification of a stereo microscope, and the numbers of larvae released were counted, collected, and preserved in 90% ethanol for genomic DNA isolation and further multiplex PCR analysis.

### 2.4. DNA Isolation

Genomic DNA was extracted from the microscopically selected individuals and pooled (10–30) larvae by crude method as per the Canadian Food Inspection Agency (CFIA) laboratory, Saskatoon, Canada. Briefly, ethanol was evaporated from the preserved larvae by air drying. Larvae were carefully washed with nuclease-free water to remove the traces of ethanol, followed by a final wash with PCR buffer (1x containing 1.5 mM MgCl_2_). Total nucleic acid was isolated from individual larvae in 20 *µ*l and pooled larvae in 50 *µ*l of PCR buffer by incubating at 90°C for 10 min, followed by immediate cooling on ice for 5 min. Larvae in buffer were adjusted with 5 *µ*l of proteinase K (10 mg/ml) and lysed at 48°C for overnight in a water bath. Proteinase K was inactivated at 90°C for 10 min. The tube was centrifuged at 10,000 × *g* for 10 min. The obtained nucleic acid was either used immediately or stored at −20°C for use within 10 days.

### 2.5. Multiplex *Trichinella* PCR Assay

The oligonucleotide primers specific to *Trichinella* species were adopted from the guidelines of the European Union Reference Laboratory for Parasites (Table 2). The oligonucleotide primers were synthesized by Eurofins Pvt. Ltd.

The PCR reaction was carried out in a total reaction volume of 30 *µ*l containing 15 *µ*l master mix (GoTaq green, Promega), 0.25 *µ*l each of forward and reverse primers (50 *µ*M) except for the cp IV primer pair targeting ITS2 region, which was 0.5 *µ*l each, 9.5 *µ*l nuclease-free water and 5 *µ*l DNA template. The amplification condition for multiplex PCR was set as predenaturation at 95°C for 4 min, with 35 amplification cycles (denaturation at 94°C for 30 s, annealing at 55°C for 60 s, and elongation at 72°C for 2 min) and a final elongation at 72°C for 10 min. After amplification, 10 *µ*l of the amplified product was subjected to 1.5% agarose gel electrophoresis and gel documentation. The obtained amplicons were observed for the presence or absence of product sizes for different *Trichinella* species [26] (Table 3).

### 2.6. Sequencing and Phylogenetic Analysis

The representative purified PCR products corresponding to 127, 155, and 404 bp were purified using the PCR product purification kit (Promega, USA) as per the manufacturer's instructions. The eluted gene was sequenced in both directions from First Base Pvt. Ltd. Malaysia for confirmation of *Trichinella* species. The amplicon of 253 bp was not sequenced as the DNA of one larva amplified this product size, and the available amount of DNA was not sufficient for sequencing. The obtained sequences were checked in NCBI using NCBI blast for their relative match with the Genebank database sequences. A single complete sequence from the forward and reverse sequences for both the ITS2 region and ESV region was constructed by analyzing the sequence chromatographs using FinchTV software and nucleotide editing using BioEdit software. The final sequences of ITS2 and ESV genes were checked using NCBI Blast for an appropriate match with *Trichinella* species and submitted to the NCBI database to obtain the accession number. The phylogenetic tree was constructed with the complete sequence in Mega software (XI version) using the maximum likelihood algorithm and by employing the Tamura-Nei model with 1,000 bootstrap statistics. For observing a relatedness of the *Trichinella* species obtained in the present study with *Trichinella* species prevalent elsewhere, the ITS-2 sequences corresponding to all *Trichinella* species available in the Genebank database were included for the phylogenetic tree construction as detailed below (Table 4).

## 3. Results

### 3.1. Tissue Digestion Assay for the Recovery of *Trichinella* Larvae

**Figure 1:**
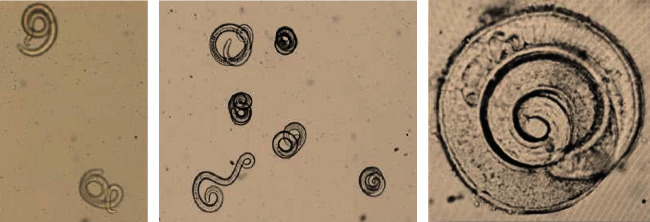
*Trichinella* larvae recovered in *Trichinella* digestion assay (10x).

The sediments of tissue digestion assay under 10x magnification of stereomicroscope revealed the presence of *Trichinella* larvae. Almost all recovered larvae were alive with rigorous motility (Figure 1). The live larvae were coiled, whereas a few dead larvae were uncoiled and comma-shaped. Overall, in this study, a total of 1,194 tongue samples were screened in 239 pooled batches (each pool with five tongue samples). Out of 239 pooled batches, *Trichinella* larvae were detected from nine batches, viz. five from Jalandhar, one from Patiala, and three from Nanital. From these nine positive batches, 15 individual samples were found positive for *Trichinella* larvae (Table 5). The density of larvae varied between one larva per 100 g and 1,290 *Trichinella* larvae per 100 g of tongue tissue samples.

Thus, the present study recorded an overall *Trichinella* positivity in pooled pig samples at the rate of 3.77% (9/239) with a positivity of 1.26% (15/1194) in individual slaughter pigs from selected places of North India. Among the four districts of Punjab, Jalandhar reported the highest positivity, 5.66% (9/159), whereas, in Uttaranchal state, the district Nainital reported a positivity of 2.22% (5/225). Further details on the sample positivity are presented in Table 5.

### 3.2. *Trichinella* PCR Assay

**Figure 2:**
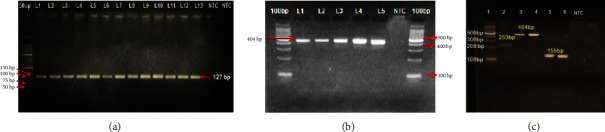
Images of agarose gel electrophoresis of PCR products: (a) larvae positive for ESV region with amplicon size of 127 bp; (b) larvae positive for ITS2 region with amplicon size of 404 bp; (c) Lane 1: 100 bp ladder, Lane 2: larvae positive for ITS1 region with amplicon size of 253 bp, Lanes 3 and 4: larvae positive for ITS2 region with amplicon size of 404 bp, Lanes 5 and 6: larvae positive for ESV region with amplicon size of 155 bp, Lane 7: no template control (NTC).

In the present study, the multiplex PCR assay did not yield the desired result. So, a single-plex PCR was carried out using the same primers individually and keeping the cycling condition as mentioned for the multiplex assay. A single-plex PCR targeting a conserved ESV region (expansion segment V region of the rRNA repeat) of the *Trichinella* genus using cpI-F and cpI-R primers amplified a product size of approximately 127 bp, whereas a product size of approximately 404 bp corresponding to the ITS2 region (internal transcribed spacer ITS2) of *T. nelsoni* was obtained with cpV-F and cpV-R primers. The DNAs of larvae were found positive for the ITS1 region of *T. britovi* with an amplicon size of approximately 253 bp with cpII-F and cpII-R primers. In the present study, recovered larvae from almost all samples were positive for the ESV (127 bp) region of genus *Trichinella*. A few larvae were positive for ESV (155 bp) and ITS2 (404 bp) region of *T. nelsoni*, whereas only one sample with five larvae was positive for 253 bp ITS1 region of *T. britovi* (Figure 2).

### 3.3. Sequencing and Phylogenetic Analysis

The obtained nucleotide sequences of the ESV region corresponding to 127 bp of the *Trichinella* genus (accession numbers PP057944 and PP057947) showed identity between 86.86% and 100% with the *Trichinella* sequences available in the NCBI database, whereas the identity of nucleotide sequences corresponding to 155 bp amplicons (accession numbers PP057945 and PP057946) varied between 89.84% and 100% in the NCBI blast search.

Nucleotide blast results of ITS-2 sequences from this study (OR607250, OR607251, OR607252, OR607253, and OR607254) matched only with nine database sequences, all of them belonging to *Trichinella* species. All ITS-2 sequences of the *Trichinella* larvae isolated from pigs in the present study showed maximum identity (percent identity 85%–86%; 99% query coverage) with *Trichinella* larvae isolated from leopard samples (accession numbers ON391046, ON391047, ON391048, ON391049, ON391050, and ON391051) and from tiger samples (accession numbers ON391052, ON391053) from IVRI, India while it matched 90.79% with ITS 2 region of *Trichinella nelson* (accession number AY851272) for which the source and place of isolation not mentioned.

For the phylogenetic analysis, in addition to the above sequences, other representative sequences of ITS-2 region for various *Trichinella* species are available in the NCBI database *viz. T. spiralis* (AY851266, AF342803, KC006415); *T. murrelli* (AY851270, KC006417, KC006414); *T. native* (AB252967, AY851267, KP307966); *T. nelson* (AY851272); *T. britovi* (AY851268, JQ403275, GU325741, KY464994); *T. patagoniensis* (MF628274); *Trichinella* T6 (AY851271, KP307971); *Trichinella* T8 (AY851273); *Trichinella* T9 (LC361216, AY851274, AB255886, LC546039); *T. zimbabwensis* (AY851276); *T. papuae* (AY851275); *T. pseudospiralis* (AY851269) were also included.

**Figure 3:**
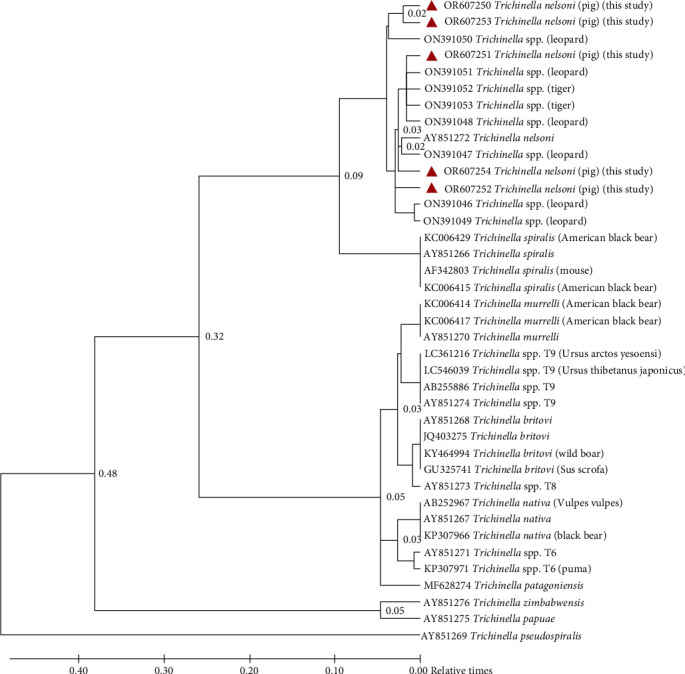
Phylogram of *Trichinella* species based on nucleotide sequences of ITS-2 region. The phylogenetic tree was constructed with the maximum likelihood algorithm to compare the relatedness of the isolated larvae (∆) with the 12 *Trichinella* species available in the Genebank database. Values of relative time for evolution are indicated on branches.

Phylogenetic analysis revealed the grouping of sequences clearly in two broad clads, the capsulated and noncapsulated *Trichinella*. Noncapsulated *Trichinella ITS-2 sequences* (AY851276; AY851275; AY851269) present as the out-group, while all capsulated *Trichinella ITS2 sequences* clustered separately into subclad, according to their species type. *ITS-2* sequences of *Trichinella* larvae recovered in the present study clustered with *Trichinella* species of IVRI (ON391046, ON391047, ON391048, ON391049, ON391050, ON391051, ON391052, and ON391053) and with *T. nelsoni* (AY851272) to form a separate subclad possibly of *T. nelsoni* within a clad of encapsulated *Trichinella* (Figure 3). *Trichinella* isolates 9.2 (OR607250) and 15.1(OR607253) were more closely related compared to the isolate nos. 9.5 (OR607251), 14.1 (OR607252), and 15.3 (OR607254).

Although the species type of *Trichinella* larvae of IVRI was not known but based on the clustering pattern with AY851272 and 404 bp amplicons of ITS-2 region corresponding to *T. nelsoni*, it is confirmed that the larvae recovered in the present study belonged to *T. nelsoni*.

## 4. Discussion and Conclusion

In India, *Trichinella* parasite has been studied very scantly which indicated low to negligible prevalence of this parasite in the country. Most studies conducted were a response to an outbreak of trichinellosis in humans. Limited studies conducted in random animals are more than 4–5 decades old [7, 13], while only two studies investigated *Trichinella* in pigs, which reported a low prevalence of 0.4%−0.6% [6, 27]. No systematic investigation was reported in India for identifying the zoonotic risk of *Trichinella* to the human population consuming pork. Of lately, there observed an increased interest in this parasite due to the potential role of pigs as a reservoir of *Trichinella* spp. and outbreaks in humans associated with pork consumption. In the recent past, few studies conducted on slaughtered pigs from Maharashtra, documented a low prevalence of 0.69% [16] and other reported absence of parasites in pigs slaughtered in Goa, Gujarat, Assam, Chandigarh, Punjab, and Uttarakhand [14, 15], indicating trichinellosis as a rare zoonosis in India.

In contrary to the previously reported studies, the present study confirms the existence of *Trichinella* spp. in slaughtered pigs in selected districts of Punjab and Uttaranchal states of North India. For the first time, zoonotic nematode larvae of *Trichinella* species have been isolated and later confirmed molecularly by PCR and sequencing. The present study reported 1.73% (10/579) and 0.81% (5/615) *Trichinella* positivity in individual pigs of Punjab and Uttaranchal, respectively, which was higher than previously reported studies from the region documenting the absence of parasite. The recorded higher positivity of 5.66% (9/159) and 2.22% (5/225) in individual slaughter pigs of Jalandhar and Nainital in the present study alarms the emergence of this neglected nematode, projecting a food safety risk. Lack of recovery of *Trichinella* larvae from slaughter pig samples from Ludhiana, Bathinda, Chandigarh, Tehri Garhwal, Pauri Garhwal, Kashipur, and Haridwar does not mean the absence of parasites, but instead, it demands a more thorough investigation with more sample size.

The molecular analysis of recovered *Trichinella* larvae in the present study confirmed the circulation of *T. nelsoni* and *T. britovi* in the region. To the best of our knowledge, this is the first study in India, which detected *Trichinella* till species level and documented the existence of *T. nelsoni* and *T. britovi* in pigs of India. Among all *Trichinella* species, *T. spiralis* is considered the classical cause of zoonotic trichinellosis in humans. However, recently, there have been increased evidences of human trichinellosis due to *T. nelsoni* and *T. britovi*. Both *T. nelsoni* and *T. britovi* are primarily the nematodes of sylvatic carnivores predominantly transmitted among wildlife in African countries and temperate areas, respectively, and with no reports from India and China [28]. Human infection with *T. britovi* is associated with the consumption of free-ranging pigs [29], whereas *T. nelsoni* has never been detected in domestic pigs but reported in bushpigs (*Potamochoerus porcus*) and warthogs (*Phacochoerus aethiopicus*) [30, 31].

No information is available on circulating *Trichinella* species in domestic or wild animals of India. Furthermore, the diagnosis of sporadic incidence of human trichinellosis is limited to the identification of the *Trichinella* genus. Detection of sylvatic *Trichinella* species in the present study highlighted the transmission of this neglected parasite between wild animal–domestic animal interfaces.

In conclusion, the recovery of live *Trichinella* larvae from slaughtered pigs intended for human consumption also raised food safety concerns in the absence of mandatory meat inspection for this parasite in the region and in the country. Upsurge in *Trichinella* positivity in slaughtered pigs is an early alarm for the emergence of foodborne risk from neglected parasitic zoonoses. Since pigs are the most important animal species as a meat source and for food security in India, the existence of these zoonotic parasites in pig production is an important public health concern that warrants extensive studies dedicated toward the epidemiological distribution and transmission of prevalent *Trichinella* genotypes in India.

## Figures and Tables

**Table 1:** Details of samples collected from the Punjab and Uttaranchal states of India.

S. no.	Name of the state	Name of district	Number of samples collected (March 2017–February 2020)
1.	Punjab	Ludhiana	133
2.		Patiala	125
3.		Jalandhar	159
4.		Bathinda	125
5.		Chandigarh	37

6.	Uttaranchal	Tehri Garhwal	126
7.		Pauri Garhwal	130
8.		Nainital	225
9.		Kashipur	07
10.		Haridwar	127
11.	Total		**1,194**

**Table 2:** Oligonucleotide primers used for the detection of *Trichinella* species.

S. no.	Target gene	Primer name	Primer sequence
1	ESV	cp-I.F	5′-GTTCCATGTGAACAGCAGT-3′
		cp-I.R	5′-CGAAAACATACGACAACTGC-3′

2	ITS1	cp-II.F	5′-GCTACATCCTTTTGATCTGTT-3′
		cp-II.R	5′-AGACACAATATCAACCACAGTACA-3′

3	ITS1	cp-III.F	5′-GCGGAAGGATCATTATCGTGTA-3′
		cp-III.R	5′-TGGATTACAAAGAAAACCATCACT-3′

4	ITS2	cp-IV.F	5′-GTGAGCGTAATAAAGGTGCAG-3′
		cp-IV.R	5′-TTCATCACACATCTTCCACTA-3′

5	ITS2	cp-V.F	5′-CAATTGAAAACCGCTTAGCGTGTTT-3′
		cp-V.R	5′-TGATCTGAGGTCGACATTTCC-3′s

**Table 3:** Amplicon sizes for different *Trichinella* species.

Name of species	*T. spiralis*	*T. nativa*	*T. britovi*	*T. pseudospiralis*	*T. murrelli*	*Trichinella* T6	*T. nelsoni*	*T. papuae*	*T. zimbabwensis*
DNA fragment size	173	127	127, 253	310 or 340 or 360	127, 316	127, 210	155, 404	240	264

**Table 4:** Details of ITS-2 sequences used for construction of the phylogenetic tree.

S. no.	Accession no.	*Trichinella* species	Place/Country	Host	Year of sequence submission
1.	OR607250 (this study)	*T. nelsoni*	Punjab, India	Pig	2023
2.	OR607251 (this study)	*T. nelsoni*	Punjab, India	Pig	2023
3.	OR607252 (this study)	*T. nelsoni*	Punjab, India	Pig	2023
4.	OR607253 (this study)	*T. nelsoni*	Uttarakhand, India	Pig	2023
5.	OR607254 (this study)	*T. nelsoni*	Uttarakhand, India	Pig	2023
6.	AY851266	*T. spiralis*	Not mentioned	Not mentioned	2011
7.	AF342803	*T. spiralis*	Washington	Mouse	2002
8.	KC006415	*T. spiralis*	Not mentioned	American black bear	2012
9.	KC006414	*T. murrelli*	Not mentioned	American black bear	2012
10.	AY851270	*T. murrelli*	Not mentioned	Not mentioned	2011
11.	KC006417	*T. murrelli*	Not mentioned	American black bear	2010
12.	AB252967	*T. native*	Japan	*Vulpes vulpes*	2008
13.	AY851267	*T. native*	Not mentioned	Not mentioned	2011
14.	KP307966	*T. native*	Not mentioned	Black bear	2015
15.	AY851272	*T. nelsoni*	Not mentioned	Not mentioned	2011
16.	AY851268	*T. britovi*	Not mentioned	Not mentioned	2011
17.	JQ403275	*T. britovi*	Not mentioned	Not mentioned	2012
18.	GU325741	*T. britovi*	Iran	*Sus scrofa*	2010
19.	KY464994	*T. britovi*	Iran	Wild boar	2017
20.	AY851276	*T. zimbabwensis*	Not mentioned	*Puma concolor*	2011
21.	MF628274	*T. patagoniensis*	Argentina	Not mentioned	2018
22.	AY851275	*T. papuae*	Not mentioned	Not mentioned	2011
23.	AY851269	*T. pseudospiralis*	Not mentioned	Not mentioned	2011
24.	AY851271	*Trichinella* T6	Not mentioned	Not mentioned	2011
25.	KP307971	*Trichinella* T6	Not mentioned	Puma	2015
26.	AY851273	*Trichinella* T8	Not mentioned	Not mentioned	2011
27.	LC361216	*Trichinella* T9	Japan	*Ursusarctos yesoensi*	2018
28.	AY851274	*Trichinella* T9	Not mentioned	Not mentioned	2011
29.	AB255886	*Trichinella* T9	Japan	Not mentioned	2006
30.	LC546039	*Trichinella* T9	Iran	*Ursus thibetanus japonicas*	2020
31.	ON391051	*Trichinella_spp*.	IVRI India	Leopard	2022
32.	ON391047	*Trichinella_spp*.	IVRI India	Leopard	2022
33.	ON391053	*Trichinella_spp*.	IVRI India	Tiger	2022
34.	ON391052	*Trichinella_spp*.	IVRI India	Tiger	2022
35.	ON391046	*Trichinella_spp*.	IVRI India	Leopard	2022
36.	ON391048	*Trichinella_spp*.	IVRI India	Leopard	2022
37.	ON391049	*Trichinella_spp*.	IVRI India	Leopard	2022
38.	ON391050	*Trichinella_spp*.	IVRI India	Leopard	2022

**Table 5:** Result of artificial digestion of pig tissue samples from Punjab and Uttaranchal (India) examined for detection of *Trichinella* larvae.

S. no.	Name of the state	District	Samples collected	Pooled batches positive	Individual pigs positive
1.	Punjab	Ludhiana	133	—	—
2.		Patiala	125	1	1
3.		Jalandhar	159	5	9
4.		Bathinda	125	—	—
5.		Chandigarh	37	—	—

6.	Uttaranchal	Tehri Garhwal	126	—	—
7.		Pauri Garhwal	130	—	—
8.		Nainital	225	3	5
9.		Kashipur	07	—	—
10.		Haridwar	127	—	—
11.	Total		**1,194**	**9**	**15**

## Data Availability

The nucleotide sequences of accession numbers used to create phylogenetic tree in the present study are available at https://blast.ncbi.nlm.nih.gov/Blast.cgi. These accession numbers are cited at relevant places within the text. Further information is available from the corresponding author.

## Additional Points

*Impact*. (1) This study reported the first evidence of sylvatic *T. britovi* and *T. nelsoni* in slaughtered pigs in India, indicating the possible transmission at the wild animal–domestic animal interface. (2) *Trichinella* positivity in slaughtered pigs is an early alarm for the emergence of foodborne risk from neglected parasitic zoonoses. (3) Isolation of numerous live *Trichinella* larvae from slaughtered pigs intended for human consumption raised emerging public health concerns for foodborne trichinellosis.
